# Synergistic Antibacterial Activity of Silver-Loaded Graphene Oxide towards *Staphylococcus Aureus* and *Escherichia Coli*

**DOI:** 10.3390/nano10020366

**Published:** 2020-02-20

**Authors:** Truong Thi Tuong Vi, Selvaraj Rajesh Kumar, Jong-Hwei Su Pang, Yu-Kuo Liu, Dave W. Chen, Shingjiang Jessie Lue

**Affiliations:** 1 Department of Chemical and Materials Engineering, Chang Gung University, Taoyuan City 33302, Taiwan; truongthituongvi005@gmail.com (T.T.T.V.); rajeshkumarnst@gmail.com (S.R.K.); ykliu@mail.cgu.edu.tw (Y.-K.L.); 2 Graduate Institute of Clinical Medical Sciences, Chang Gung University, Taoyuan City 33302, Taiwan; jonghwei@mail.cgu.edu.tw; 3 Department of Orthopedic Surgery, Chang Gung Memorial Hospital, Keelung City 20445, Taiwan; 4 Department of Safety, Health and Environment Engineering, Ming-Chi University of Technology, New Taipei City 24301, Taiwan

**Keywords:** antibacterial activity, graphene oxide, silver, synergistic, reactive oxygen species

## Abstract

In this study, the physicochemical and surface properties of the GO–Ag composite promote a synergistic antibacterial effect towards both Gram-negative *Escherichia coli* (*E. coli*) and Gram-positive *Staphylococcus aureus* (*S. Aureus*) bacteria. GO–Ag NPs have a better bactericidal effect on *E. coli* (73%) and *S. Aureus* (98.5%) than pristine samples (pure Ag or GO). Transmission electron microscopy (TEM) confirms that the GO layers folded entire bacteria by attaching to the membrane through functional groups, while the Ag NPs penetrated the inner cell, thus damaging the cell membrane and leading to cell death. Cyclic voltammetry (CV) tests showed significant redox activity in GO–Ag NPs, enabling good catalytic performance towards H_2_O_2_ reduction. Strong reactive oxygen species (ROS) in GO–Ag NPs suggests that ROS might be associated with bactericidal activity. Therefore, the synergy between the physicochemical effect and ROS production of this material is proposed as the mechanism of its antibacterial activity.

## 1. Introduction

Bacterial infections and disease are common and are often treated with antibiotics, but the advent of multidrug resistance in pathogenic bacteria has decreased the effectiveness of antibiotics [1]. Frequent and inappropriate usage of conventional antibiotics has led to reduced effectiveness in clinical treatment. Therefore, to complement the conventional antibiotic treatments, the development of bactericidal nanomaterials has become important [2] because the mode of action/mechanism for the nanoparticle (NPs) is direct contact with the bacterial cell wall, which promotes antibacterial activity [3].

Graphene is a two-dimensional material that consists of a single layer of sp^2^ carbon atoms [4,5]. Graphene oxide (GO) and reduced GO contain functional groups and a unique structure of the carbon basal plane, which contribute to its antibacterial activity. Recently, GO-based materials were used to enhance antibacterial performance and showed 88–100% bacterial inhibition at dosages of 100–300 µL mL^−1^ [6]. GO demonstrated the most powerful antibacterial activity towards *Escherichia coli* (*E. coli*) among graphene-based materials [7]. GO directly interacts with the bacterial membrane and causes membrane stress [8]. Tu et al. showed that pristine graphene nanosheets reduced *E. coli* viability by destroying the extraction of phospholipid bacterial membranes [9]. Therefore, GO derivatives are considered to be a new weapon for combatting multidrug resistance bacteria while reducing toxicity to normal human cells [6,10].

Silver nanoparticles (Ag NPs) have attracted much attention because of their effectiveness in biological applications due to a high surface-area-to-volume ratio [11,12]. The benefits of Ag NPs include multiple mechanisms for bactericidal effect, such as binding with bacteria DNA, blocking energy recycling, and releasing Ag^+^ ion [13,14,15]. The Ag NPs mechanism was proven to produce the thermal effect via hyperthermia production and promote long-term antibacterial action, thereby reducing the Gram-negative and Gram-positive bacteria population [16,17]. However, Ag NPs tend to agglomerate and are prone to oxidization, thus limiting the antibacterial effect. For instance, Ag NPs were found to be poorly dispersed in biological solution when grafted onto a titanium or silica substrate [18,19,20,21]. To overcome this problem, combining GO with Ag NPs facilitates the antibacterial properties. GO sheets can act as a substrate to stabilize and prevent Ag NPs from agglomeration and supply enhancement for antimicrobial activity.

There are many linkers and reducing agents used in GO–Ag NP fabrication. For example, hydroquinone and poly(diallyl dimethyl ammonium chloride) have been reported as linkers [22,23] but they are known as toxic chemicals. Das et al. prepared different ratios of sodium borohydride (NaBH_4_) in the presence of trisodium citrate acting as a stabilizing agent. Highly dense 2–25 nm Ag NPs were obtained. Yet, the reducing agent or stabilizer role has not been well-derived [24]. Tai et al. prepared a 0.05 M AgNO_3_ precursor solution to fabricate GO–Ag NPs using poly(acrylic acid) as both linker and reducing agent. The final GO–Ag NPs product contained 16.2% Ag contents, indicating a low Ag formation rate and therefore the antibacterial inhibition zone was low (9.9 to 11 mm) [25]. Slawomir et al. used poly(vinyl alcohol) as a stabilizer and formed large size (80–230 nm) Ag NPs in GO sheets [26]. The obtained Ag NP sizes in GO–Ag NP product tend to be larger than 10 nm without using linkers [23,27,28,29]. Therefore, choosing good linkers to optimize the bactericidal effect is essential. Chitosan (CS) is an eco-friendly and biocompatible material that exhibits a bactericidal property [30]. CS was well-known because it contributed to enhancing the antimicrobial activity against many bacteria, fungi, and yeast [31,32,33]. The CS antibacterial activity contribution is due to intrinsic factors such as the positive charge, molecular weight, hydrophilicity, or the soluble ionic solution, etc. [34]. Previous studies showed that optimizing the CS/GO/Ag ratio enhanced the antibacterial effect. For instance, Marta et al. prepared GO/Ag composites grafted with CS to evaluate the bactericidal effect towards methicillin-resistant *Staphylococcus aureus* (*S. Aureus*) [35]. Khawaja et al. proved that enhanced CS coating with GO–Ag NPs improved the inhibition zone (19–22 mm) compared with that of GO–Ag NPs only (15–18 mm) [36]. However, the Ag NPs’ size effect before and after modifying with GO or the key factors between the three GO, Ag NPs, and CS components have not been well-discussed. Furthermore, the deeper mechanism about the morphology before and after treatment with bacteria, the reactive oxygen species production, the role of pure components in the response to the antibacterial effect and the synergistic effect have been poorly investigated in previous studies [22,23,28,29,37]. We note that those factors should be examined together to reveal the whole antibacterial mechanism process.

In previous studies, we demonstrated that GO–Ag NPs exhibited good bactericidal activity by applying NaSH as the intermediate bridge to orient Ag NPs onto GO sheet. However, the mechanism has not mentioned [38]. To better understand the bactericidal activity and improve the mechanism, a novel approach to fabricate GO–Ag NPs using the reducing agent sodium borohydride and linker CS was introduced in this study. The antibacterial activity effect was evaluated on *E. coli* as Gram- negative bacterium and on *S. Aureus* as Gram-positive bacterium. Using the growth curve inhibition test and disk diffusion assay, a comparison between the nanocomposite and pure component was conducted. In addition, the detailed mechanism between GO–Ag and the bactericidal effect was proposed via morphological observation and reactive oxygen species to correlate to the antibacterial activity.

## 2. Materials and Methods

### 2.1. Materials

Graphite powder (G), potassium bromide (KBr), hydrogen peroxide (H_2_O_2_), chitosan 448869, acetic acid, Hoechst 333,242 (HS), propidium iodide (PI), glutaraldehyde (50%), osmic acid (4%), Nafion solution, 2′,7′-dichlorodihydroflourescein diacetate (DCFA-DA), and dimethyl sulfoxide (DMSO) were purchased from Sigma-Aldrich, St. Louis, MO, USA. Potassium permanganate (KMnO_4_) and trisodium citrate (Na_3_C_6_H_5_O_7_) were purchased from Nihon Shiyaku Industries Ltd., Osaka, Japan. Sulfuric acid (H_2_SO_4_, 95–98%) solution was purchased from Scharlab S.L., Barcelona, Spain. Silver nitrate (AgNO_3_) was purchased from Mallinckrodt Baker Inc., Paris, France, and hydrochloric acid (HCl) was purchased from Showa Chemical Co., Ltd., Honshu, Japan.

### 2.2. Bacterial Strains

*S. Aureus* (BCRC 10781) and *E. coli* (DH5α) were obtained from the Bioresource Collection and Research Center in Hsinchu, Taiwan. Luria–Bertani (LB) broth, agar–agar powder and phosphate-buffered saline (PBS) were purchased from Sigma-Aldrich (St. Louis, MO, USA).

### 2.3. GO Synthesis

GO was synthesized via the modified Hummer’s method based on our previous work with slight modification [39]. In brief, 2 g graphite powder was dissolved in 300 mL H_2_SO_4_ with continuous stirring for 10 min. Two grams of KMnO_4_ was added. After the green color of the solution changed to a black color, six portions of additional KMnO_4_ (2 g) were sequentially added. Until MnO^3−^ was completely oxidated, 400 g ice was added into the solution which was placed in the an ice bath to reduce the reaction temperature. The solution was held for a few days until separation of precipitation was clearly observed. The upper solution was discarded, and the remaining precipitate was washed with HCl and deionized (DI) water via centrifugation (Hitachi, Tokyo, Japan) until the pH reached neutral. The gel-like product was dried in a 60 °C vacuum oven overnight.

### 2.4. Ag NPs Synthesis

Ag NPs were synthesized according to the previous study [40]. First, 2 mL of 12.5 mM trisodium citrate was added into 5 mL of 0.375 mM AgNO_3_. An aliquot of H_2_O_2_ was added, followed by addition of 2.5 mL of NaBH_4_. An amount of 45 µL KBr was added with continuous stirring, until a yellow color was observed in the solution, indicating that the Ag NPs were formed. The solution was held in the dark and stored at room temperature for further analysis. Solutions can be stored for up to 4 months. To collect the powder product, the solution was centrifuged at 10,000 rpm and dried under vacuum for further analysis.

### 2.5. GO–Ag NP Synthesis

GO–Ag NPs were synthesized according to a previous study, with slight modification [35]. An amount of 0.1 g GO was sonicated in 50 mL DI water for 20 min until completely exfoliated. An amount of 20 mL GO suspension was added into 5 mL of 0.375 mM AgNO_3_. Subsequenlty, 2.5 mL of NaBH_4_ was added in the presence of 5 mL chitosan in 1% (*w*/*v*) acetic acid solution. The mixture was continuously stirred for 4 h. The solution was filtered using dialysis tubing to remove the unreacted Ag. The products were collected after several washes with DI water, centrifuged, and dried under 60 °C vacuum overnight.

### 2.6. Characterizations

Two-dimensional images were observed using transmission electron microscopy (TEM, JEM 2000EXII, JEOL, Tokyo, Japan). To compare the distribution of the particle size with the total counts, ImageJ software was used to analyze the full width at half maximum (FWHM).

The prepared samples were analyzed using X-ray diffraction (XRD, model D5005D, Siemens AG, Munich, Germany) to examine the crystal structure. The diffractogram was recorded at 2θ ranging from 5 to 80° at a scan rate of 4°/min with Cu Kα radiation. Fourier transform infrared spectroscopy (FT-IR) (model Horiba FT-730, Minami-ku, Kyoto, Japan) was used to evaluate the functional groups and chemical bonding of GO and GO–Ag NPs. Samples were scanned in the range of 800–4000 cm^−1^. A UV–visible spectrophotometer (V-650, Jasco, Tokyo, Japan) was applied to measure the light transmittance. The color was reflected in the absorption wavelength. X-ray photoelectron spectroscopy (Thermo Scientific™ K-Alpha™ XPS, Thermo Fisher Scientific, Waltham, MA, USA) was applied to examine the chemical composition of the samples. Zeta potential were recorded in triplicate using a dynamic laser scattering analyzer (Zetasizer, 2000 HAS, Malvern, Worcestershire, UK) at room temperature. Subsequently, the mass loss and the weight of the sample contents were detected using a thermogravimetric analyzer (TGA 2050, TA instrument, Inc., Tokyo, Japan).

### 2.7. Cyclic Voltammetry Measurement

Cyclic measurements were collected with a conventional three-electrode cell containing silver/silver chloride (Ag/AgCl) as the reference electrode and a platinum electrode (1 × 1 cm) as the counter electrode. Prior to testing, GO–Ag NPs and GO were sonicated sequentially in isopropanol and deionized water for approximately 20 min. A 5 wt % Nafion solution was used as a binder and was added to the mixture. These mixtures were sprayed on carbon cloth using a spray gun with an amount of 1 mg for each sample. Phosphate buffer solution (PBS, 0.01 M, pH 7.0) was mixed with H_2_O_2_ to obtain the final concentration of 1 mM H_2_O_2_ solution. The solution including PBS and H_2_O_2_ were used as the supporting electrolyte, and high-purity nitrogen gas was bubbled for 20 min prior to starting the experiments. Cyclic voltammetry (CV) tests were conducted from −0.2 to 0.8 V at a scan rate of 50 mV s^−1^.

### 2.8. Antibacterial Test

#### 2.8.1. Bacterial Growth Curve Assay

Initially, *S. Aureus* and *E. coli* were grown overnight in Luria Broth (LB) under aerobic conditions at 37 °C using a shaker incubator. The bacterial suspension was diluted in LB to achieve an optical density of 0.3 at 600 nm wavelength (OD 600 nm). The bacterial suspensions were transferred into 48-well plates. Following this procedure, 50 µL of each sample was added (pure LB was used as a control sample) to 450 µL LB, and the samples were placed in a shaking incubator for 1 h. Subsequently, 10 µL of sample mixture was removed and transferred into 96-well plates, and 90 µL LB medium was added. All treatments were performed in triplicate and measured by a microplate reader (Biotek, Hong Kong, China) with an optical density at wavelength 600 nm (OD 600 nm) within 5 h. The inhibition efficiency was calculated using the following equation

(1) $$\mathrm{Inhibition}\;(\%)=\left(1-\frac{\Delta \;OD\;sample\;treatment}{\Delta OD\;control}\;\right)\times 100$$

#### 2.8.2. Disk Diffusion Assay

In brief, a 100 μL bacteria suspension (OD 600 nm was 0.1) was spread on petri dishes containing agar and LB medium. The agar plates were prepared in 90 mm petri dishes with 22 mL of agar medium giving a final depth of 3 mm. Wells (9 mm in diameter) were punched in the culture media with 100 μL of samples diluted in DI water to obtain final concentration of 100, 50, and 10 μg mL^−1^. The plates were incubated at 37 °C for 24 h. Antimicrobial activity was assessed by measuring the diameter of the growth-inhibition zone in millimeters (including the well diameter of 9 mm) for the bactericidal test.

#### 2.8.3. Change in Bacterial Morphology after Sample Exposure

TEM analysis was applied to investigate the bacterial morphologies before and after treatments. In detail, *E. coli* and *S. Aureus* bacteria were grown overnight and diluted to reach an of 0.1 at OD 600 nm. The bacteria were suspended in 100 μg mL^−1^ GO, Ag NPs, and GO–Ag NPs. The mixture was incubated at 37 °C for 2 h. Bacteria without treatment were used as a control sample. Subsequently, 10 µL of bacteria solution was transferred onto the 24-well plates, and the samples were fixed with 2.5% glutaraldehyde for 1 h at room temperature. The samples were washed twice with PBS before postfixing with 1% osmic acid for 45 min. Finally, the samples were again washed twice with PBS and subjected to increasing gradients (30%, 50%, 70%, and 100%) of ethanol for 10 min. The samples were drop cast onto a TEM gold grid before testing.

#### 2.8.4. Live/Dead Cell Staining Using Confocal Laser Scanning Microscopy

Confocal laser scanning microscopy (Zeiss LSM 510-Meta- Heidelberg-Germany) was used to evaluate the bacterial colony population. The bacterial suspension was diluted in PBS solution (to obtain an OD value equal to 0.15). Bacterial suspensions were separately treated with 100 μg mL^−1^ GO, Ag NPs, and GO–Ag NPs for 2 h. The solution was mixed with HS dye as a live cell indicator and PI dye as a dead cell indicator at a ratio of 5:1 (HS:PI). The bacterial suspension was incubated for 20 min and washed twice using PBS solution. Before it was observed under a confocal laser scanning microscope (CLSM).

#### 2.8.5. Reactive Oxygen Species Mechanism Applied to Bacteria Treatment

The production of ROS was analyzed using the sensitive dye 2′,7′-dichlorodihydroflourescein diacetate (DCFA-DA). In the experiment, a stock solution of 10 mM was prepared in DMSO solution. Approximately 100 µL of bacterial suspension (OD~0.1) was impregnated with 100 μg mL^−1^ GO, Ag NPs and GO–Ag NPs for 2 h incubation. The mixture was twice washed using PBS to remove the medium. Subsequently, 100 µL DCFH-DA of 25 µM was added and incubated for 30 min in the dark at 37 °C. Prior to testing, the bacteria were washed using PBS to remove residual dye. Fluorescence images were captured using a CLSM at an excitation of 485 nm and emission of 535 nm.

## 3. Results

### 3.1. Synthesis of GO, Ag NPs, and GO–Ag NPs

**Figure 1:**
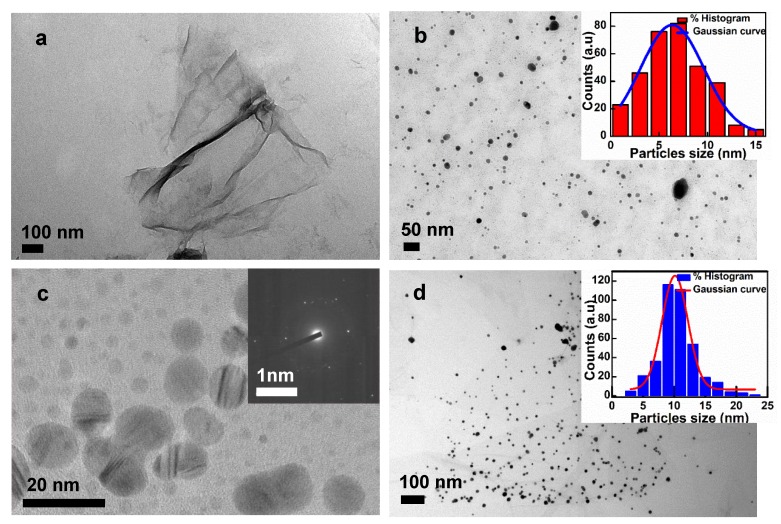
TEM images of GO (**a**), Ag NPs along with the its particle size distribution (**b**), HRTEM image of Ag NPs at 500 kV with electron diffraction (**c**), and TEM image of GO–Ag NPs with Ag particle size distribution (**d**). Size distribution analysis was analyzed using ImageJ software. (IJ152-NIH Image, Research Service Branch, National Institute of Mental Health, Bethesda, MD, USA).

**Scheme 1:**
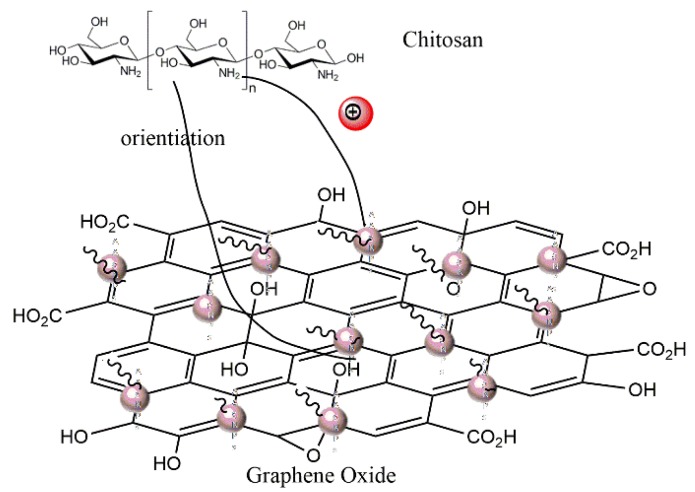
GO-Ag NPs formation in the presence of CS as a linker, orientating Ag NPs into the GO’s matrix.

The TEM analysis was used to observe pristine GO, Ag NPs, and GO–Ag NPs composite morphologies. As indicated in Figure 1a, pristine GO showed a flaky, smooth and paper-like structure with a size range >1 µm. Figure 1b showed that Ag NPs were observed with a small spherical shape. The particle size of Ag NPs was calculated by ImageJ software with a mean size of 7.4 nm and fitted to a Gaussian curve, which is indicated in the insect of Figure 1b. Figure 1c shows the HRTEM image and insect of Figure 1c represents a selected area electron diffraction of Ag NPs. As expected, the Ag NPs have a single orientation [41]. The TEM images showed the migration of Ag NPs anchored on GO, as shown in Figure 1d. Ag NPs were uniformly distributed on the surface of the GO layers. The Ag NP size distribution was calculated with a mean size of approximately 10.1 nm and was fitted with a Gaussian curve (insect of Figure 1d). Compared with pure Ag NPs, the Ag NP size in GO–Ag NPs was increased to 30%. The difference in Ag NP size in GO–Ag NPs can be proposed as follows: when the precursor Ag NO_3_ meets an acidic solution an Ag ion phase was formed, and therefore Ag^+^ ions probably bound to chitosan macromolecules via electrostatic interaction between the electron–rich oxygen atoms of the CS polar hydroxyl and ether groups and the electropositive transition cations. Meanwhile, the hydroxyl and amino groups from chitosan can interact, forming hydrogen bonding with hydroxyl, carbonyl, and carboxyl moieties in the graphene oxide sheets. This can orient Ag NPs to the GO’s matrix (Scheme 1). The influence of the low molecular weight CS and the experiment was carried out at room temperature, therefore its effect on the increase in Ag NPs size during promoting nucleation [42,43,44].

**Figure 2:**
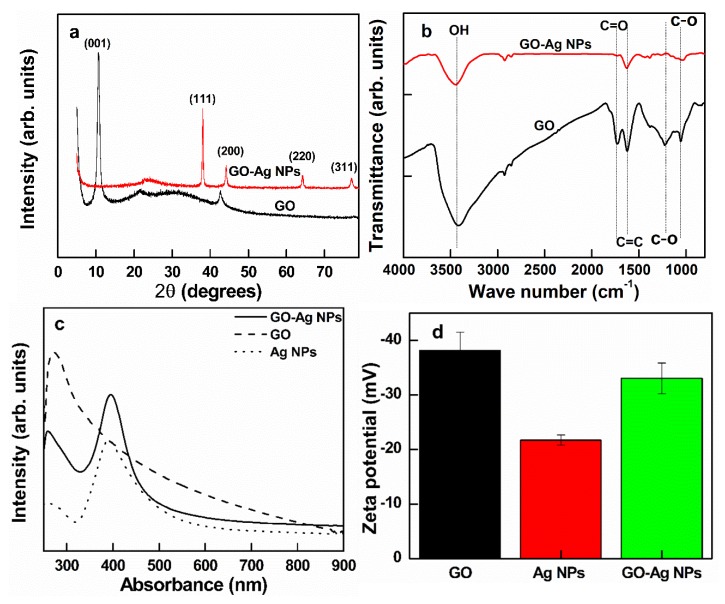
XRD patterns (**a**) and FTIR spectra (**b**) of GO and GO–Ag NPs; UV spectra of GO, Ag NPs and GO–Ag NPs (**c**); Zeta potential profiles of pure GO, Ag NPs, and GO–Ag NPs (**d**). The error bars in (**d**) represent the standard deviations of the data performed in triplicate (*n* = 3).

The binding of Ag NPs onto GO was confirmed by XRD (Figure 2a) and FTIR analysis (Figure 2b). As shown in Figure 2a, the GO exhibited a typical peak at 11.7° with d spacing at 7.8 Å. After modifying with Ag NPs, GO–Ag NPs composite shifted the main XRD peaks to 38.1°, 44.3°, 64.5°, and 77.5°, which are assigned to the (111), (200), (220), and (311) crystal lattice planes of face-centered cubic Ag NPs, respectively. However, the typical GO peak at 11.7° disappeared in the GO–Ag samples due to Ag NPs attached to the interlayers and covering the signals of the GO peaks [45]. Besides, the FTIR in Figure 2b results proved the characteristic of the domain groups from GO and GO–Ag NPs. The peaks of the characteristic GO spectrum were observed at 3404, 1733, 1622, 1220, and 1055 cm^−1^ and these spectral peaks were associated with the stretching vibration of C–OH (hydroxyl) and C=O, the vibration of C=C (possibly due to the skeletal vibration of oxidized graphite domains) and C–O and stretched C–O, respectively. The presence of oxygen-containing groups such as carboxyl, hydroxyl and epoxy groups confirmed the oxidation process of GO. The corresponding FTIR peaks were similarly matched with those of a previous study [39]. As compared with GO, most of those functional groups of the GO peaks significantly decreased in GO–Ag NPs. This result occurs because the interaction between Ag NPs and GO decrease the intensity signal [46]. Obviously, the disappearance of the groups C=O and C–O at 1733 cm^−1^ and 1220 cm^−1^ suggests that Ag NPs were bonded to GO via those groups [47].

As indicated in Figure 2c, the pristine GO exhibited a typical peak at 230 nm and a shoulder near 310 nm corresponding to *π–π* * electronic transition of C=C aromatic bonds and *n–π* * electronic transition of C=O bonds. The UV–visible data showed that Ag NPs exhibited a sharp peak at a wavelength of 400 nm, indicating successfully synthesized Ag NPs [48]. The UV–visible data showed that GO–Ag NPs have two main peaks: one peak near 230 nm from GO and the other peak near 400 nm from Ag NPs. The zeta potential profiles were describle in Figure 2d. As shown in Figure 2d, UV–visible profiles showed GO had a negative zeta potential near −38 mV, indicating a moderately stable dispersion whereas Ag NPs had lower value near −21 mV. As expected, the mid-point value nearly −33 mV confirmed that GO–Ag NPs was successfully synthesized.

**Figure 3:**
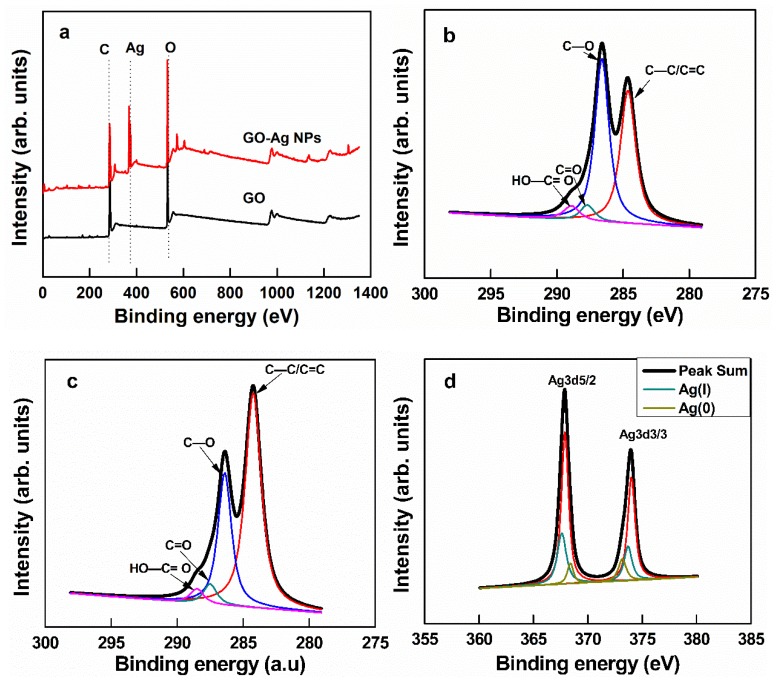
Full scan XPS spectra of GO and GO–Ag NPs (**a**), XPS spectra for C1s deconvolution of GO (**b**), XPS spectra for C1s deconvolution of GO–Ag NPs (**c**), and XPS spectra for Ag 3d deconvolution of GO–Ag NPs (**d**).

The elemental compositions of GO and GO–Ag NPs were examined using XPS analysis, as shown in Figure 3a. The full scan spectra of GO showed a C/O ratio near 2.3:1, which matched with the literature data [49]. Wide scan analysis revealed that the Ag element peak was found in GO–Ag NPs (Figure 3a). According to the C1s deconvolution XPS spectra of the main GO bonding groups, the unique peaks at 284.4, 285.8, 287, and 288.5 eV corresponded to C–C, C–O, C=O and O–C=O groups, respectively (Figure 3b). During the silver attachment process, the C–C bonding ratio was relatively increased, while the oxygen groups ratio was decreased [46] (Figure 3c). Details of the functional groups are described in Table 1. Moreover, Figure 3d displays the high-resolution XPS spectrum of the Ag3d region in the nanocomposite. Two main peaks were observed at 368.04 eV and 373.54 corresponding to Ag3d5/2 and Ag3d3/2. The peaks in the Ag(I) state were higher than those in the Ag (0) state.

**Figure 4:**
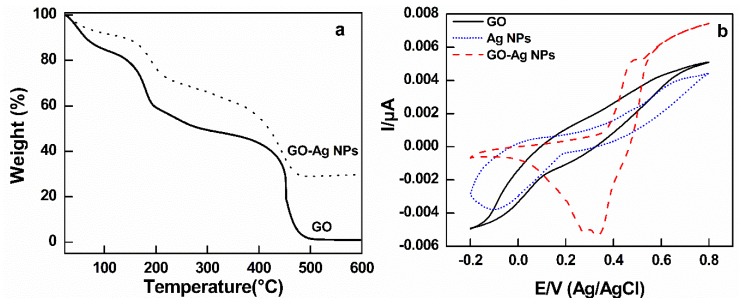
TGA analysis of GO and GO–Ag NPs in air atmosphere at the heating rate of 5 °C min^−1^ (**a**) and CV curves for GO, Ag NPs and GO–Ag NPs (**b**). The CV test was carried out in 0.01 M PBS and 1 mM H_2_O_2_ electrolyte at the scan rate of 50 mV s^−1^.

TGA analysis indicated that pristine GO was degraded in the air atmosphere (Figure 4a). The GO exhibited three stages of weight loss. The first peak decreased from 25 to 100 °C due to the removal of water from the remaining moisture. Other noticeable weight loss peaks occurred at 150– 250 °C and 400–500 °C. The former peak near 180 °C is ascribed to the removal of the oxygen functional groups from the GO surface, and the other sharp peak near 450 °C is related to the burning of the carbon constituting the graphene sheets. TGA measurement was used to evaluate the Ag NPs weight percentage in GO–Ag composites. Because the Ag NPs are stable at a high temperature of 1000 °C and the complete degradation of GO at 490 °C, thereby the leftover Ag content was determined by the weight loss of the GO–Ag NPs. Therefore, the weight percentage of Ag NPs was estimated approximately 30% in GO–Ag NPs.

### 3.2. Cyclic Voltammetry Test

The cyclic voltammetry (CV) test is a reliable tool for enhancing ROS measurement in the presence of H_2_O_2_ [50]. This method has been associated with cell cycle arrest, apoptosis, migration, and inflammation and antibacterial activity. Thereby, the CV test of H_2_O_2_ plays an important role in identifying the biological and pathological mechanism systems [51]. According to Figure 4b, no peak was detected in the GO sample, whereas a small reduction peak near −0.1 mV was found in Ag NPs, indicating that Ag NPs have a potential ROS response [52]. Moreover, GO–Ag NPs exhibited a prominent potential of 0.33 V towards the reduction of H_2_O_2_. This synergistic behavior of GO–Ag NPs was not present in GO or Ag NPs. This method offers new biological insights into early bacterial stimulation of the antibacterial response and supplies a new tool for investigating free radical scavenging of materials [53].

### 3.3. Antibacterial Test

#### 3.3.1. Diffusion Disk Assay

**Figure 5:**
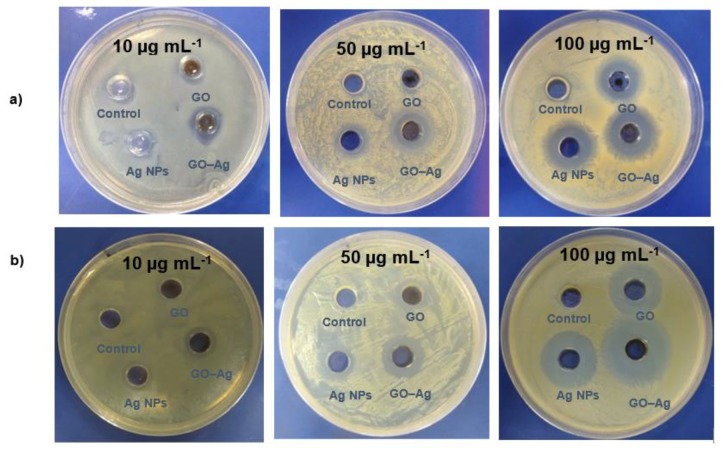
Digital images of antibacterial activity using disk diffusion assay for control, GO, Ag NPs, and GO–Ag NPs toward *E. coli* (**a**) and *S. Aureus* (**b**) after treatment with nanomaterials. From left to right, the concentrations were gradually increased from 10, 50, to 100 µg mL^−1^, respectively.

The zone of inhibition analysis is illustrated in Figure 5 and Table 2. Figure 5a displays *E. coli* zone inhibition. After treatment with pure Ag NPs and GO alone at concentration of 10 µg mL^−1^, the *E. coli* disk showed a small inhibition zone (9.5 mm). In contrast, the GO–Ag NP zone was 11 mm wider than that of pristine Ag and GO. At 50 µg mL^−1^, the inhibition zone dimensions were 10, 11, and 13 mm for GO, Ag NPs, and GO–Ag NPs, respectively. With increasing concentration to 100 µg mL^−1^, the zone inhibition became obviously wider with values of 15, 16, and 18 mm for GO, Ag NPs, and GO–Ag NPs, respectively.

Figure 5b displays *S. Aureus* zone inhibition. It can be observed that the zone inhibition after treatment with pure GO and Ag NPs was almost the same as that with *E. coli* at a concentration 10 µg mL^−1^. After treatment with GO–Ag NPs, the inhibition zone of *S. Aureus* (11.5 mm) was slightly wider than that of *E. coli* (11 mm). At 50 µg mL^−1^, the zone diameter value were 10.5, 11.5, and 15 mm corresponding to GO, Ag NPs, GO–Ag NPs, respectively.

At a concentration of 100 µg mL^−1^, the inhibition zone dimension was more obvious after treatment with pure GO and Ag NPs. The zone diameter value were 18 mm and 20 mm corresponding to GO and Ag NPs. The largest zone inhibition was found for treatment with GO–Ag NPs (28 mm). This result demonstrated that the antibacterial activity showed synergistic performance for combination of Ag with GO and zone inhibition after treatment with GO–Ag NPs. In this test, *S. Aureus* was more susceptible than *E. coli*.

#### 3.3.2. Bacterial Growth Curve Inhibition Assay

**Figure 6:**
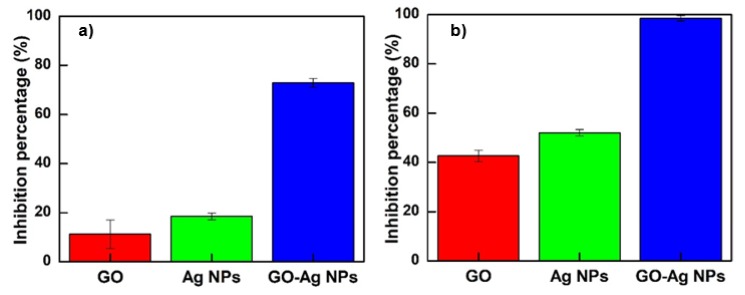
Bacterial inhibition percentage (%) of GO, Ag NPs, and GO–Ag NPs towards *E. coli* (**a**) and *S. Aureus* (**b**) for 5 h incubation at the same concentration of 100 µg mL^−1^. The results are expressed as the means ± standard deviation of three independent experiments.

Growth curve analysis of the 600 nm optical density (OD 600 nm) was used to determine the inhibition percentage towards *E. coli* (Figure 6a) and *S. Aureus* (Figure 6b) using GO, Ag NPs and GO–Ag NPs. The antimicrobial inhibition percentage increased steadily from GO, Ag NPs to GO–Ag NPs. The quantitative inhibition percentage was only 11% and 18% for GO and Ag NPs towards *E. coli* but increased to 43% and 52% for *S. Aureus*, respectively. Moreover, the antibacterial effect significantly increased to 73% towards *E. coli* and 98.5% towards *S. Aureus* for GO–Ag NPs. In agreement with the disk diffusion assay, the GO–Ag NPs exhibited the most efficient bactericidal effect. These results also confirm that *S. Aureus* was more susceptible than *E. coli*.

#### 3.3.3. Morphology Before and After Treatment

**Figure 7:**
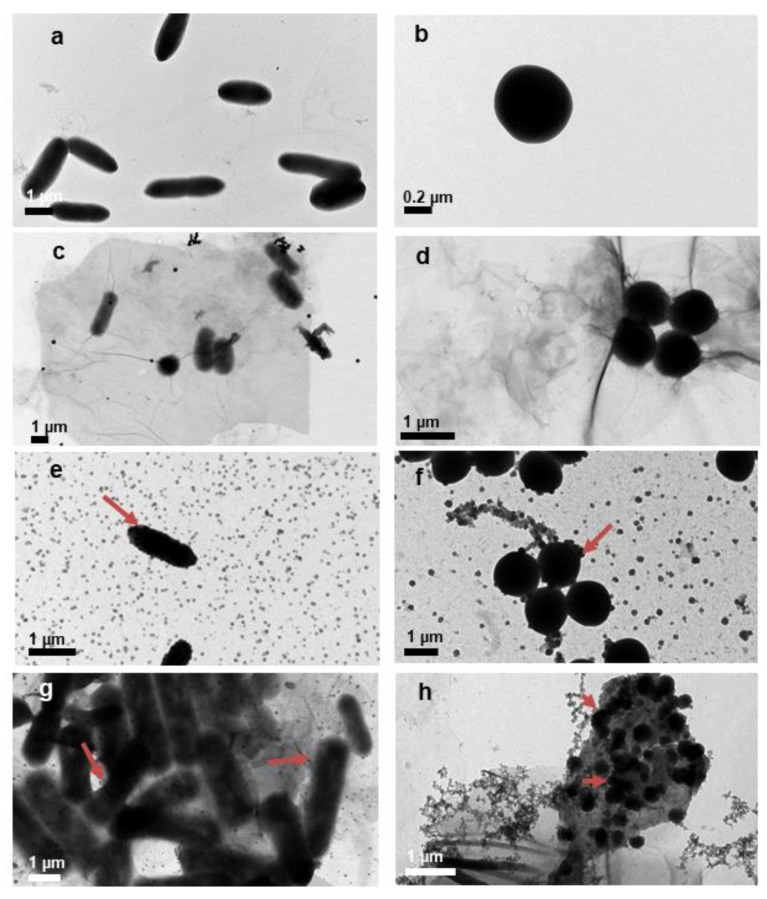
TEM images of pristine *E. coli* (**a**) and *S. Aureus* (**b**), those exposed to GO (**c**,**d**), to Ag NPs (**e**,**f**), and to GO-Ag NPs (**g**,**h**), respectively. Red arrows indicate the penetration of Ag NPs into the bacterial cell.

The TEM micrographs showed that compared with the control groups, most of the bacterial cells lost shape integrity when treated with nanomaterials (Figure 7). The *E. coli* control group exhibited the typical rod shape (Figure 7a), whereas *S. Aureus* appeared as a spherical shape (Figure 7b). After treatment with GO, *E. coli* and *S. Aureus* were physically wrapped in GO sheets, and the outer bacterial membrane was damaged (Figure 7c,d). The Ag NPs can directly contact the bacterial cell membrane by penetrating or accumulating onto the outer membrane (as indicated by the red arrow in Figure 7e,f). After treatment with GO–Ag NPs, bacterial cells had the most severe damage, loss of shape structure, and deformation after treatment, which might be caused by the loss of intracellular contents (Figure 7g,h). Thus, the *E. coli* and *S. Aureus* morphologies showed more significant damage after treatment with GO–Ag NPs than with pure GO and Ag NPs.

#### 3.3.4. Live/Dead Cell Staining

**Figure 8:**
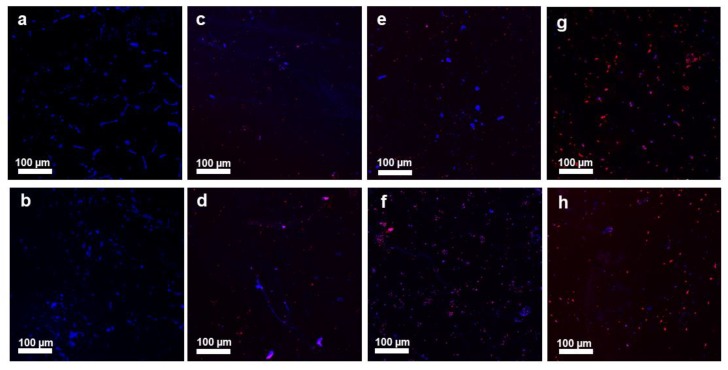
Confocal fluorescent images of control *E. coli* (**a**) and control *S. Aureus* (**b**), those exposed to GO (**c**,**d**), to Ag NPs (**e**,**f**), and to GO-Ag NPs (**g**,**h**), respectively. The fluorescent images are captured under CLSM after dying with HS and PI.

Confocal microscopy was used to visualize the quantity of bacterial colonies before and after nanomaterials treatment. The images of *E. coli* without treatment showed the presence of a high number of live bacteria, as shown in Figure 8a. After treatment with GO, most of the bacteria were still alive, and only a low number of dead bacteria was found in Figure 8c. A larger number of dead bacterial cells was observed after treatment with Ag NPs, as indicated in Figure 8e. In contrast, most of the dead *E. coli* were observed in GO–Ag NPs (Figure 8g). Figure 8b,d,f,h shows the results for *S. Aureus* bacteria. As indicated in Figure 8d, the number of dead bacteria treated with GO was higher than that in the control *S. Aureus* (Figure 8b). The ratio of dead *S. Aureus* treated with Ag NPs (Figure 8f) was higher than that treated with GO. The largest number of dead bacteria was found after treatment with GO–Ag NPs (Figure 8h). Evidently, the ratio of dead *S. Aureus* after treated with GO, Ag, and GO–Ag NPs, respectively was higher than that of *E. coli*.

#### 3.3.5. Reactive Oxygen Species Mechanism Applied for Bacterial Treatment

**Figure 9:**
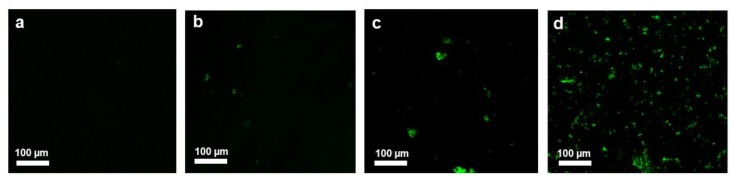
Untreated *E. coli* without observable fluorescence (**a**), changes of ROS production after treatment of GO (**b**), after treatment with Ag NPs (**c**), and after treatment with GO-Ag NPs (**d**), respectively. The fluorescent images are captured under CLSM after dying with DCFH-DA (green indicator).

DCFH-DA is a well-known nonpolar dye that is converted into the polar derivative DCFH when oxidized by intracellular ROS [54]. The fluorescence images of the control and treated samples after background subtraction are shown in Figure 9a–d. The control sample (Figure 9a) showed almost no green color. GO and Ag NPs exhibited week green fluorescence (Figure 9b,c) and produced low ROS. However, noticeably increased green fluorescence was observed in *E. coli* treated with GO–Ag NPs, indicating that silver-loaded graphene oxide produced a significant ROS level (Figure 9d).

## 4. Discussion

Spherical Ag NPs doped with GO were prepared to evaluate their bactericidal effect and compared with pristine components. The results showed that the GO–Ag NPs composites exhibited an excellent impact on the antibacterial property. Although the graphene-based Ag literature has reported on antibacterial activity (Table 3), the mechanism and the interplay of GO and Ag NPs have been rarely discussed. In this study, we hypothesis that physical and chemical factors impacted the antibacterial activity.

The controversies related to the GO antibacterial effect still remain unanswerable. Recent research has also indicated that GO was proven to have non-antibacterial activity. The factors influence to antibacterial effect come the medium culture condition (Mueller–Hinton Broth) or the purity of GO [55]. In contrast, other reports showed that GO had a bactericidal effect [10,56]. Previously, a three-step antibacterial mechanism of graphene-based materials was proposed: initial cell deposition, membrane stress caused by direct contact and oxidation stress on glutathione, which serve as a redox mediator in bacteria [7]. Our results supporting those articles showed that GO is a biological macromolecule that exhibits an antibacterial effect. Herein, as shown in Figure 7c,d. *E. coli* and *S. Aureus* were adhered on GO layers, and partial shape deformation occurred. Therefore, physical attack is one factor that influences the antibacterial activity. It is well known that the hydrophobicity of the interlayers sp^2^ in GO creates a strong interaction between GO sheets and the lipid bilayer of the bacterial cell membranes [57]. Therefore, the GO wrapping process on bacteria occurs via a physical mechanism. Elfremova et al. proved that GO had an antibacterial effect by neutralizing the bacterial surface charge [8]. Sengupta et al. found that antibacterial activity occurred via physical wrapping with the bacterial membrane, whereas Liu et al. proposed that GO contained functional groups that might form much more stable dispersions, thus offering more opportunities to interact with cells for cell deposition [7]. Moreover, the sharp edges of GO sheets allow it to attach to the cell membrane, and the consequential integrity disruption is the major cause of membrane damage [58,59].

For Ag NPs, it was reported that Ag can interact with sulfur-containing proteins from the cell membrane and phosphorus-containing compounds in the cells, attacking the respiratory chain with cell division and leading to cell death [13]. Thus, direct contact with Ag NPs or exposure to a high concentration of Ag ions occurs easily, producing elevated antibacterial activity. Cationic nanoparticles could induce nanoscale holes, and this phenomenon could be caused by interactions between cationic nanoparticles and the cell membrane [19]. However, Ag NPs might aggregate or big Ag NPs production reduced of active specific area. Our result obtained Ag NPs with a small size of 7.4 nm (insect of Figure 1b). It was noted that the small-size Ag NP surface-area-to-volume ratio for individual particles increases and the relative particle concentration also increases [48,60]. According to the TEM images of the *E. coli* and *S. Aureus* morphology after treatment with Ag NPs (Figure 7e and 7f), certain Ag NPs accumulate in the membrane, while others penetrate the inner cell and kill the bacteria (Ag NPs are indicated by red arrow).

**Figure 10:**
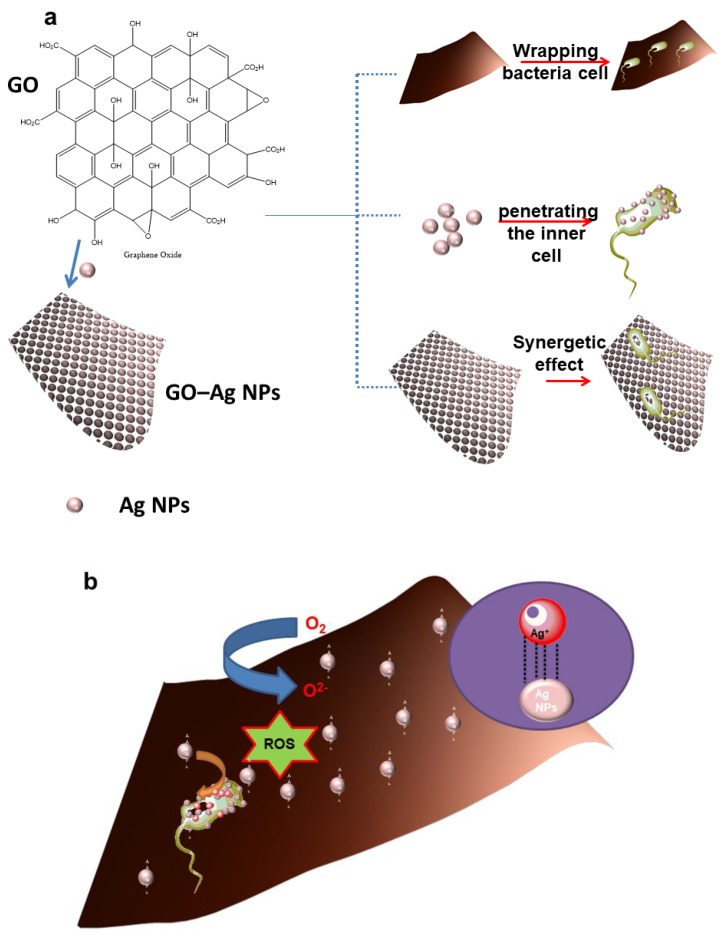
Physical attack by bacteria of Ag, GO and GO–Ag NPs (**a**) and ROS inducement as one of the important factors for the bactericidal effect of GO–Ag NPs (**b**).

For GO–Ag NPs, the rapid and excellent antibacterial activities of the nanocomposites were attributed to the synergistic effect of GO nanosheets and Ag NPs (illustrated in Figure 10a). These results are in agreement with those of other works [38,45,61]. In TEM images of the bacterial morphology after treatment by GO–Ag NPs (Figure 7g,h), both *E. coli* and *S. Aureus* were more severely damaged as a cluster colony compared with those that received pure GO or Ag NPs treatment. However, the good physicochemical properties of GO–Ag NPs support their antibacterial activity. The physicochemical property represented by the surface charge, which plays a key role in bacterial adhesion. Compared with Ag NPs, GO–Ag NPs exhibited a higher zeta potential value, which indicates better stability, suggesting higher antibacterial activity. This result was in agreement with that of Zhao et al., who prepared Ag NPs incorporated with GO that enhanced stability, significantly weakened the viability of bacteria, and improved the long-term antibacterial activity [62]. In the presence of CS, it was noted that CS has a positive charge, which enhances bonding with the negatively charged bacterial cell wall and therefore leads to larger cell damage [35,36]. By possessing CS as a stabilizer-mediated chemical, the Ag NPs assumed a well-ordered arrangement on the GO sheets [63]. On the other hand, in the presence of CS, note that CS has a positive charge which enhances the bonding with negatively charged bacterial cell walls and therefore leads to larger cell damage [36]. Moreover, the covalent bond between CS and GO could improve the dispersion, thereby resulting in better aqueous solubility with enhanced antimicrobial activity [64,65]. The resulting GO–Ag NPs obtained by CS grafting with the Ag NPs size was nearly 10 nm, smaller than that of 50–70 nm Ag NPs using sodium citrate as a stabilizer [29]. Compared with the scattering Ag NPs on GO sheets prepared by Bao et al. [23], the Ag NPs were well-decorated on the GO layers due to the CS linker role into the GO’s matrix in this research. TEM images showed the Ag NPs were more regularly arranged onto GO sheets (Figure 1d) than that of in pristine Ag NPs without GO substrate (Figure 1b). After conjugation with GO, Ag NPs did not show a dramatic change in size. The inset of Figure 1d shows that the size distribution of Ag particles in GO–Ag NPs was 10.1 nm, slightly larger than that of Ag particles (7.4 nm). According to previous study, Shekhar Agnihotri et al. proved that Ag NPs did not exhibit a significant difference in antibacterial effect in the range 5–10 nm [40]. Besides, Figure 4a showed TGA results revealed that obtained Ag NPs contents in GO–Ag NPs was approximately 30%, higher than that prepared by Tai et al. (16.2% Ag NPs) [25]. As shown in the XPS data results (Figure 3d), the two peaks were deconvoluted into 367.68 and 373.69 eV (representing the Ag(I) state), higher than those peaks at 368.53 and 373.17 eV (representing the Ag (0) state). According to a previous study, it was proved that high Ag(I) promotes high antibacterial activity performance [66].

In addition, the reactive oxygen stress (ROS) also plays an important role in antibacterial properties, as shown in Figure 4b and Figure 9. It is noted that induced oxidative stress is caused by the accumulation of intracellular ROS, such as hydrogen peroxide, superoxide anions, hydroxyl radicals, and singlet molecular oxygen [67]. The CV test was conducted using PBS as the supporting electrolyte in the presence of H_2_O_2_ electrolyte, which attracted much interest as an indicator of oxidative stress [50,53]. As indicated in Figure 4b, Ag NPs displayed a shoulder peak near −0.1 V, which means that Ag NPs might exhibit potential for ROS production, whereas an apparent strong redox peak of GO–Ag NPs shifted at −0.3 V suggests a notably active surface [68]. In contrast, GO shows almost no oxidative stress (no significant peak was observed), leading to a lesser bactericidal effect. According to previous studies, the reduction in H_2_O_2_ in the CV test is associated with the more activated sites, which were decomposed or consumed, thereby leading to the imbalance for redox efflux [69,70]. The increment of the GO–Ag NPs peak to −0.3 mV compared with −0.1 mV in Ag NPs might occur because the enhancement of area surface facilitates the electron transfer process to the electrode [71]. To examine this point, DCFH-DA was applied as an ROS indicator with *E. coli* bacteria. As expected, Figure 9 displayed widespread green fluorescence after treatment with GO–Ag NPs. In contrast, for treatment with GO and Ag NPs, the green color appears as scattered dots. This result is attributed to a significant synergistic effect by the high ROS production in GO–Ag NPs.

This research also showed that Gram-positive *S. Aureus* exhibited a greater bactericidal effect than Gram-negative *E. coli*. It was proved that Gram-negative *E. coli* bacteria are generally more resistant because their outer impermeable membrane protects the cell wall peptidoglycans [72]. The thick peptidoglycan layer in Gram-positive bacteria containing lipid groups on the surface might contribute to the added interaction between Gram-positive bacteria and GO [73]. Akhavan et al. proved that by direct contact with bacteria, the sharp edge of GO, which is perpendicular to the substrate, caused more severe damage to *S. Aureus* than to *E. coli* because*S. Aureus* has no additional outer membrane [74]. Moreover, R. Bhat et al. raised the hypothesis that Gram-positive bacteria more strongly interacted with these nanomaterials through electrostatic and hydrogen bonding and physical piercing of the cell membrane, whereas Gram-negative bacteria interacted with the nanomaterials through direct physical contact only [3].

Table 3 summarizes the antibacterial effect of previously reported publications and this study. All of the reports examined the GO–Ag NPs antibacterial effect with little emphasis on the mechanism [22,23,24,25,27,28,35,36,38]. Compared with the other papers, our study has demonstrated the synergistic bactericidal activity of GO–Ag NPs. Using more than one antibacterial method of investigation, the synergistic effect of GO–Ag NPs was strongly proved. A possible synergistic effect was proposed by Figure 10a wherein GO acts as the scaffold, promoting direct attack of Ag NPs towards bacteria. The synergistic effect not only originates from the physicochemical properties but also is derived from ROS production or penetration of Ag into the inner cell (as illustrated in Figure 10b). The ROS could damage the balance of the DNA and protein function or cell division, thereby destroying and interrupting the biochemical processes, leading to cell death [75].

## 5. Conclusions

In summary, facile GO–Ag NPs synthesis using CS as an eco-friendly linker was successfully proposed via an in situ method. Both quantitative and qualitative antibacterial effects were examined via a diffusion test, bacterial growth curve inhibition assay and live/dead bacterial analysis. Compared with pure GO and Ag NPs, GO–Ag NPs exhibited the most effective inhibition towards *E. coli* (73%) and *S. Aureus* (98.5%). Under TEM image observation, the morphologies of *S. Aureus* and *E. coli* were more severely damaged by treatment with GO–Ag NPs than pristine GO and Ag NPs. The synergetic effect of GO–Ag NPs was fully proposed by multiple mechanisms, including the physicochemical effects and the ROS production. Due to the enhanced surface area, GO–Ag NPs could promote more bacterial adhesion, thus facilitating greater cell death when combined with Ag NPs and GO. In addition, ROS were confirmed by the CV test, and DCFA-DA offers an in-depth examination of the antibacterial mechanism. In summary, the synergistic GO–Ag NP antibacterial effect is beneficial for promising practical treatment of disease.

## Figures and Tables

**Table 1:** Functional group percentage (%) of chemical bonds presents in GO and GO–Ag NPs as determined from C1s deconvolution of the corresponding high-resolution XPS spectra

C1s	C=C	C–O	C=O	O–C–O
GO	42.29	48.11	4.81	4.79
GO–Ag NPs	69.60	22.70	4.79	2.61

**Table 2:** Disc diffusion inhibition zones for *S. Aureus* and *E. coli*

	Inhibition Zone (mm)					
Concentration (μg mL^−1^)	GO		Ag NPs		GO–Ag NPs	
	*E. coli*	*S. Aureus*	*E. coli*	*S. Aureus*	*E. coli*	*S. Aureus*
10	9.5	9.0	9.5	9.5	11.0	11.5
50	10.0	10.5	11.0	11.5	13.0	15.0
100	15.0	18.0	16.0	20.0	18.0	28.0

**Table 3:** Antibacterial effect of GO–Ag NPs.

Linker	Reducing Agents	Bacteria Model	Ag NPs Size	Evaluation Method	Inhibition	Compared with Pure GO or Ag NPs	Mechanism of Action	Reference
None	Hydroquinone	*E. coli* and *S. Aureus*	80 nm	- Plate counting - Disk diffusion	- 100 and 87.6% - 18 and 21 mm.	- 51.9 and 61.3% by GO - 9 and 10 mm by GO	None	[23]
None	PVP	*E. coli*, *S. Aureus*, *S. epidermidis*, and *C. Albicans*	80–230 nm	GCI	- 77.5–88.6%	- None	ROS production (by fluorescence)	[26]
None	Glucose	*E. coli*	10–70 nm	ADA	- N/A	- No antibacterial effect by GO	Morphology observation	[28]
None	Sodium citrate	*P. aeruginosa*	7.5 nm	ADA	- 100%	- No antibacterial effect by GO	None	[37]
None	Sodium citrate	*E. coli* and *S. Aureus*	50–70 nm	GCI	- 60% for GCI both bacteria strain - ADA: 74% towards *S. Aureus* and 94% towards *E. coli*	- No antibacterial effect by GO and Ag NPs	Morphology observation, cell division disorder.	[29]
None	NaBH_4_	*S. Aureus* and *B. subtilis*	2–25 nm	GCI and plate counting	- 100%	- 96–97% by Ag NPs	None	[24]
PAA	None	*E. coli* and *S. Aureus*	4–8 nm	Disk diffusion	- 9.9 and 11.4 mm	- N/A	None	[25]
PDDA	Sodium citrate	*E. coli* and B. subtilis	14 nm	ADA	- 54 and 60%	- No antibacterial effect by Ag NPs	None	[22]
STPP	CS	*E. coli* and *S. aureus*	20 ± 1 nm	Disk diffusion	- 18.5 and 17 mm	- 5 and 6 mm	None	[27]
NaSH	NaSH	*S. Aureus* and *P. aeruginosa*	2 nm	GCI	- 100%	- 27.2% and 32. 7% induced by GO	None	[38]
CS	Green tea leaves	*S. Aureus*, *S. mutans*, *E. coli, K. pneumoniae, P. aeruginosa, S. typhi*	15–25 nm	Disk diffusion	- 19–22 mm	- 7–11 mm by GO	None	[36]
CS	NaBH_4_	*S. Aureus*	10–30 nm	MBC and MIC	- 1.09 and 4.05 µg mL^−1^	- 100%	None	[35]
CS	NaBH_4_	*E. coli* and *S. Aureus*	10.1 nm	GCI and disk diffusion	- 73 and 98.5% - 28 and 18 mm.	- 11 and 18% by GO; 43 and 52 by Ag NPs. - 15 and 18 mm by GO; 16 and 20 mm by Ag NPs	Morphology observation, ROS production (by CV test and fluorescence)	This study

**Notes:** ADA: agar diffusion assay; GCI: growth curve inhibition; PDDA: poly (diallyl dimethyl ammonium chloride); PAA: poly (acrylic acid); STPP: sodium tripolyphosphate; PVP: poly(diallyl dimethyl ammonium chloride); *S. epidermidis*: *Staphylococcus epidermidis*; *C. albicans*: *Candidas albicans*; *S. mutans*: *Streptococcus mutans*; *K. pneumoniae*: *Klebsiella pneumoniae*; *S. typhi*: *Salmonella typhi*; *B. subtilis*: *Bacillus subtilis*; *P. aeruginosa*: *Pseudomonas aeruginosa*; N/A: not applicable (mentioned but not shown clear results). None: did not test.

## References

[1] van der Meer J.W.M. (2013). The infectious disease challenges of our time. Front. Public Health.

[2] Gupta A., Mumtaz S., Li C.-H., Hussain I., Rotello V.M. (2019). Combatting antibiotic-resistant bacteria using nanomaterials. Chem. Soc. Rev..

[3] Wang L., Hu C., Shao L. (2017). The antimicrobial activity of nanoparticles: Present situation and prospects for the future. Int. J. Nanomed..

[4] Geim A.K., Novoselov K.S. (2007). The rise of graphene. Nat. Mater..

[5] Kapitanova O.O., Emelin E.V., Dorofeev S.G., Evdokimov P.V., Panin G.N., Lee Y., Lee S. (2020). Direct patterning of reduced graphene oxide/graphene oxide memristive heterostructures by electron-beam irradiation. J. Mater. Sci. Technol..

[6] Kumar P., Huo P., Zhang R., Liu B. (2019). Antibacterial Properties of Graphene-Based Nanomaterials. Nanomaterials.

[7] Liu S., Zeng T.H., Hofmann M., Burcombe E., Wei J., Jiang R., Kong J., Chen Y. (2011). Antibacterial Activity of Graphite, Graphite Oxide, Graphene Oxide, and Reduced Graphene Oxide: Membrane and Oxidative Stress. ACS Nano.

[8] Efremova L.V., Vasilchenko A.S., Rakov E.G., Deryabin D.G. (2015). Toxicity of Graphene Shells, Graphene Oxide, and Graphene Oxide Paper Evaluated with *Escherichia coli* Biotests. Biomed. Res. Int..

[9] Tu Y., Lv M., Xiu P., Huynh T., Zhang M., Castelli M., Liu Z., Huang Q., Fan C., Fang H. (2013). Destructive extraction of phospholipids from *Escherichia coli* membranes by graphene nanosheets. Nat. Nanotechnol..

[10] Yousefi M., Dadashpour M., Hejazi M., Hasanzadeh M., Behnam B., de la Guardia M., Shadjou N., Mokhtarzadeh A. (2017). Anti-bacterial activity of graphene oxide as a new weapon nanomaterial to combat multidrug-resistance bacteria. Mater. Sci. Eng. C.

[11] Zhang X.-F., Liu Z.-G., Shen W., Gurunathan S. (2016). Silver Nanoparticles: Synthesis, Characterization, Properties, Applications, and Therapeutic Approaches. Int. J. Mol. Sci..

[12] Abou El-Nour K.M.M., Eftaiha A.A., Al-Warthan A., Ammar R.A.A. (2010). Synthesis and applications of silver nanoparticles. Arab. J. Chem..

[13] Wong K.K.Y., Liu X. (2010). Silver nanoparticles—The real “silver bullet” in clinical medicine?. Med. Chem. Commun..

[14] Dakal T.C., Kumar A., Majumdar R.S., Yadav V. (2016). Mechanistic Basis of Antimicrobial Actions of Silver Nanoparticles. Front. Microbiol..

[15] Zafar N., Shamaila S., Nazir J., Sharif R., Shahid Rafique M., Ul-Hasan J., Ammara S., Khalid H. (2016). Antibacterial Action of Chemically Synthesized and Laser Generated Silver Nanoparticles against Human Pathogenic Bacteria. J. Mater. Sci. Technol..

[16] D’Agostino A., Taglietti A., Desando R., Bini M., Patrini M., Dacarro G., Cucca L., Pallavicini P., Grisoli P. (2017). Bulk Surfaces Coated with Triangular Silver Nanoplates: Antibacterial Action Based on Silver Release and Photo-Thermal Effect. Nanomaterials.

[17] D’Agostino A., Taglietti A., Grisoli P., Dacarro G., Cucca L., Patrini M., Pallavicini P. (2016). Seed mediated growth of silver nanoplates on glass: Exploiting the bimodal antibacterial effect by near IR photo-thermal action and Ag^+^ release. RSC Adv..

[18] López-Heras M., Theodorou I.G., Leo B.F., Ryan M.P., Porter A.E. (2015). Towards understanding the antibacterial activity of Ag nanoparticles: Electron microscopy in the analysis of the materials-biology interface in the lung. Environ. Sci. Nano.

[19] Slavin Y.N., Asnis J., Häfeli U.O., Bach H. (2017). Metal nanoparticles: Understanding the mechanisms behind antibacterial activity. J. Nanobiotechnol..

[20] Ghilini F., Rodríguez González M.C., Miñán A.G., Pissinis D., Creus A.H., Salvarezza R.C., Schilardi P.L. (2018). Highly Stabilized Nanoparticles on Poly-l-Lysine-Coated Oxidized Metals: A Versatile Platform with Enhanced Antimicrobial Activity. ACS Appl. Mater. Interfaces.

[21] Pallavicini P., Dacarro G., Taglietti A. (2018). Self-Assembled Monolayers of Silver Nanoparticles: From Intrinsic to Switchable Inorganic Antibacterial Surfaces. Eur. J. Inorg. Chem..

[22] Zhu Z., Su M., Ma L., Ma L., Liu D., Wang Z. (2013). Preparation of graphene oxide–silver nanoparticle nanohybrids with highly antibacterial capability. Talanta.

[23] Bao Q., Zhang D., Qi P. (2011). Synthesis and characterization of silver nanoparticle and graphene oxide nanosheet composites as a bactericidal agent for water disinfection. J. Colloid Interface Sci..

[24] Das M.R., Sarma R.K., Borah S.C., Kumari R., Saikia R., Deshmukh A.B., Shelke M.V., Sengupta P., Szunerits S., Boukherroub R. (2013). The synthesis of citrate-modified silver nanoparticles in an aqueous suspension of graphene oxide nanosheets and their antibacterial activity. Colloids Surf. B Biointerfaces.

[25] Tai Z., Ma H., Liu B., Yan X., Xue Q. (2012). Facile synthesis of Ag/GNS-g-PAA nanohybrids for antimicrobial applications. Colloids Surf. B Biointerfaces.

[26] Jaworski S., Wierzbicki M., Sawosz E., Jung A., Gielerak G., Biernat J., Jaremek H., Łojkowski W., Woźniak B., Wojnarowicz J. (2018). Graphene Oxide-Based Nanocomposites Decorated with Silver Nanoparticles as an Antibacterial Agent. Nanoscale Res. Lett..

[27] Rasoulzadehzali M., Namazi H. (2018). Facile preparation of antibacterial chitosan/graphene oxide-Ag bio-nanocomposite hydrogel beads for controlled release of doxorubicin. Int. J. Biol. Macromol..

[28] Ma J., Zhang J., Xiong Z., Yong Y., Zhao X.S. (2011). Preparation, characterization and antibacterial properties of silver-modified graphene oxide. J. Mater. Chem..

[29] Tang J., Chen Q., Xu L., Zhang S., Feng L., Cheng L., Xu H., Liu Z., Peng R. (2013). Graphene Oxide—Silver Nanocomposite As a Highly Effective Antibacterial Agent with Species-Specific Mechanisms. ACS Appl. Mater. Interfaces.

[30] No H.K., Young Park N., Ho Lee S., Meyers S.P. (2002). Antibacterial activity of chitosans and chitosan oligomers with different molecular weights. Int. J. Food Microbiol..

[31] Roller S., Covill N. (1999). The antifungal properties of chitosan in laboratory media and apple juice. Int. J. Food Microbiol..

[32] Fernandes J.C., Tavaria F.K., Soares J.C., Ramos O.S., Joao Monteiro M., Pintado M.E., Xavier Malcata F. (2008). Antimicrobial effects of chitosans and chitooligosaccharides, upon Staphylococcus aureus and *Escherichia coli*, in food model systems. Food Microbiol..

[33] Lopez-Moya F., Lopez-Llorca L. (2016). Omics for Investigating Chitosan as an Antifungal and Gene Modulator. J. Fungi..

[34] Kong M., Chen X.G., Xing K., Park H.J. (2010). Antimicrobial properties of chitosan and mode of action: A state of the art review. Int. J. Food Microbiol..

[35] Marta B., Potara M., Iliut M., Jakab E., Radu T., Lucaci F., Gabriel K., Popescu O., Astilean S. (2015). Designing chitosan-silver nanoparticles-graphene oxide nanohybrids with enhanced antibacterial activity against Staphylococcus aureus. Colloids Surf. A Physicochem. Eng. Asp..

[36] Khawaja H., Zahir E., Asghar M.A., Asghar M.A. (2018). Graphene oxide, chitosan and silver nanocomposite as a highly effective antibacterial agent against pathogenic strains. Colloids Surf. A Physicochem. Eng. Asp..

[37] de Faria A.F., Martinez D.S.T., Meira S.M.M., de Moraes A.C.M., Brandelli A., Filho A.G.S., Alves O.L. (2014). Anti-adhesion and antibacterial activity of silver nanoparticles supported on graphene oxide sheets. Colloids Surf. B Biointerfaces.

[38] Vi T.T.T., Rajesh Kumar S., Rout B., Liu C.-H., Wong C.-B., Chang C.-W., Chen C.-H., Chen D.W., Lue S.J. (2018). The Preparation of Graphene Oxide-Silver Nanocomposites: The Effect of Silver Loads on Gram-Positive and Gram-Negative Antibacterial Activities. Nanomaterials.

[39] Baskoro F., Wong C.-B., Kumar S.R., Chang C.-W., Chen C.-H., Chen D.W., Lue S.J. (2018). Graphene oxide-cation interaction: Inter-layer spacing and zeta potential changes in response to various salt solutions. J. Membr. Sci..

[40] Agnihotri S., Mukherji S., Mukherji S. (2014). Size-controlled silver nanoparticles synthesized over the range 5–100 nm using the same protocol and their antibacterial efficacy. RSC Adv..

[41] Ravulapalli S., Mariserla B.M.K., Soma V.R., Saritha R., Rao D. (2010). Biosynthesis of Silver Nanoparticles Using Coriandrum Sativum Leaf Extract and Their Application in Nonlinear Optics. Adv. Sci. Lett..

[42] Regiel A., Irusta S., Kyzioł A., Arruebo M., Santamaria J. (2012). Preparation and characterization of chitosan-silver nanocomposite films and their antibacterial activity against Staphylococcus aureus. Nanotechnology.

[43] Honary S., Ghajar K., Khazaeli P., Shalchian P. (2011). Preparation, Characterization and Antibacterial Properties of Silver-Chitosan Nanocomposites Using Different Molecular Weight Grades of Chitosan. Trop. J. Pharm. Res..

[44] Hien N., Phu D., Duy N., Quoc L., Lan N., Hoang D., Van H., Nu P., Thai Hoa T. (2015). Influence of Chitosan Binder on the Adhesion of Silver Nanoparticles on Cotton Fabric and Evaluation of Antibacterial Activity. Adv. Nanopart..

[45] Yang Y.-K., He C., He W.-J., Yu L.-J., Peng R.-G., Xie X.-L., Wang X.-B., Mai Y.W. (2011). Reduction of silver nanoparticles onto graphene oxide nanosheets with N, N-dimethylformamide and SERS activities of GO/Ag composites. J. Nanopart. Res..

[46] Çiplak Z., Yildiz N., Çalimli A. (2014). Investigation of Graphene/Ag Nanocomposites Synthesis Parameters for Two Different Synthesis Methods. Fuller. Nanotub. Carbon Nanostructures.

[47] Ţucureanu V., Matei A., Avram A.M. (2016). FTIR Spectroscopy for Carbon Family Study. Crit. Rev. Anal. Chem..

[48] Raza M.A., Kanwal Z., Rauf A., Sabri A.N., Riaz S., Naseem S. (2016). Size- and Shape-Dependent Antibacterial Studies of Silver Nanoparticles Synthesized by Wet Chemical Routes. Nanomaterials.

[49] Al-Gaashani R., Najjar A., Zakaria Y., Mansour S., Atieh M.A. (2019). XPS and structural studies of high quality graphene oxide and reduced graphene oxide prepared by different chemical oxidation methods. Ceram. Int..

[50] Rawson F.J., Hicks J., Dodd N., Abate W., Garrett D.J., Yip N., Fejer G., Downard A.J., Baronian K.H.R., Jackson S.K. (2015). Fast, Ultrasensitive Detection of Reactive Oxygen Species Using a Carbon Nanotube Based-Electrocatalytic Intracellular Sensor. ACS Appl. Mater. Interfaces.

[51] Yang D., Ni N., Cao L., Song X., Alhamoud Y., Yu G., Zhao J., Zhou H. (2019). Silver Doped Mesoporous Silica Nanoparticles Based Electrochemical Enzyme-Less Sensor for Determination of H_2_O_2_ Released from Live Cells. Micromachines.

[52] Chen Y.C., Hsu J.H., Hsu Y.K. (2018). Branched silver nanowires on fluorine-doped tin oxide glass for simultaneous amperometric detection of H_2_O_2_ and of 4-aminothiophenol by SERS. Mikrochim. Acta.

[53] Rudzka D.A., Cameron J.M., Olson M.F. (2015). Reactive oxygen species and hydrogen peroxide generation in cell migration. Commun. Integr. Biol..

[54] Rastogi R.P., Singh S.P., Hader D.P., Sinha R.P. (2010). Detection of reactive oxygen species (ROS) by the oxidant-sensing probe 2′, 7′-dichlorodihydrofluorescein diacetate in the cyanobacterium Anabaena variabilis PCC 7937. Biochem. Biophys. Res. Commun..

[55] Barbolina I., Woods C.R., Lozano N., Kostarelos K., Novoselov K.S., Roberts I.S. (2016). Purity of graphene oxide determines its antibacterial activity. Materials.

[56] Anand A., Unnikrishnan B., Wei S.-C., Chou C.P., Zhang L.-Z., Huang C.-C. (2019). Graphene oxide and carbon dots as broad-spectrum antimicrobial agents—A minireview. Nanoscale Horiz..

[57] Romero-Vargas Castrillón S., Perreault F., de Faria A.F., Elimelech M. (2015). Interaction of Graphene Oxide with Bacterial Cell Membranes: Insights from Force Spectroscopy. Environ. Sci. Technol. Lett..

[58] Hui L., Piao J.-G., Auletta J., Hu K., Zhu Y., Meyer T., Liu H., Yang L. (2014). Availability of the Basal Planes of Graphene Oxide Determines Whether It Is Antibacterial. ACS Appl. Mater. Interfaces.

[59] Sengupta I., Bhattacharya P., Talukdar M., Neogi S., Pal S.K., Chakraborty S. (2019). Bactericidal effect of graphene oxide and reduced graphene oxide: Influence of shape of bacteria. Colloid Interface Sci. Commun..

[60] Samberg M.E., Orndorff P.E., Monteiro-Riviere N.A. (2011). Antibacterial efficacy of silver nanoparticles of different sizes, surface conditions and synthesis methods. Nanotoxicology.

[61] Gao L., Wang L., Yang L., Zhao Y., Shi N., An C., Sun Y., Xie J., Wang H., Song Y. (2019). Preparation, characterization and antibacterial activity of silver nanoparticle/graphene oxide/diatomite composite. Appl. Surf. Sci..

[62] Zhao R., Lv M., Li Y., Sun M., Kong W., Wang L., Song S., Fan C., Jia L., Qiu S. (2017). Stable Nanocomposite Based on PEGylated and Silver Nanoparticles Loaded Graphene Oxide for Long-Term Antibacterial Activity. ACS Appl. Mater. Interfaces.

[63] Jiang Y., Gong J.-L., Zeng G.-M., Ou X.-M., Chang Y.-N., Deng C.-H., Zhang J., Liu H.-Y., Huang S.-Y. (2016). Magnetic chitosan–graphene oxide composite for anti-microbial and dye removal applications. Int. J. Biol. Macromol..

[64] Li P., Gao Y., Sun Z., Chang D., Gao G., Dong A. (2016). Synthesis, Characterization, and Bactericidal Evaluation of Chitosan/Guanidine Functionalized Graphene Oxide Composites. Molecules.

[65] Yang X., Tu Y., Li L., Shang S., Tao X.-M. (2010). Well-Dispersed Chitosan/Graphene Oxide Nanocomposites. ACS Appl. Mater. Interfaces.

[66] Chook S.W., Chia C.H., Zakaria S., Ayob M.K., Chee K.L., Huang N.M., Neoh H.M., Lim H.N., Jamal R., Rahman R. (2012). Antibacterial performance of Ag nanoparticles and AgGO nanocomposites prepared via rapid microwave-assisted synthesis method. Nanoscale Res. Lett..

[67] Schieber M., Chandel N.S. (2014). ROS function in redox signaling and oxidative stress. Curr. Biol..

[68] Geetha Bai R., Muthoosamy K., Shipton F.N., Pandikumar A., Rameshkumar P., Huang N.M., Manickam S. (2016). The biogenic synthesis of a reduced graphene oxide-silver (RGO–Ag) nanocomposite and its dual applications as an antibacterial agent and cancer biomarker sensor. RSC Adv..

[69] Fu L., Lai G., Mahon P., Wang J., Zhu D., Jia B., Malherbe F., Yu A. (2014). Carbon Nanotube and Graphene Oxide Directed Electrochemical Synthesis of Silver Dendrites. RSC Adv..

[70] Jones A.M., Garg S., He D., Pham A.N., Waite T.D. (2011). Superoxide-Mediated Formation and Charging of Silver Nanoparticles. Environ. Sci. Technol..

[71] Lakshmi V., Balavijayalakshmi J. (2018). Silver Nanocomposites Decorated Reduced Graphene Oxide Nanosheets for Electrochemical Sensor Applications. Orient. J. Chem..

[72] Wimmerstedt A., Kahlmeter G. (2008). Associated antimicrobial resistance in *Escherichia coli*, *Pseudomonas aeruginosa*, *Staphylococcus aureus*, *Streptococcus pneumoniae* and *Streptococcus pyogenes*. Clin. Microbiol. Infect..

[73] Pulingam T., Thong K.L., Ali M.E., Appaturi J.N., Dinshaw I.J., Ong Z.Y., Leo B.F. (2019). Graphene oxide exhibits differential mechanistic action towards Gram-positive and Gram-negative bacteria. Colloids Surf. B Biointerfaces.

[74] Akhavan O., Ghaderi E. (2010). Toxicity of Graphene and Graphene Oxide Nanowalls Against Bacteria. ACS Nano.

[75] Cabiscol E., Tamarit J., Ros J. (2000). Oxidative stress in bacteria and protein damage by reactive oxygen species. Int. Microbiol. Off. J. Span. Soc. Microbiol..

